# Roles and therapeutic potential of different extracellular vesicle subtypes on traumatic brain injury

**DOI:** 10.1186/s12964-023-01165-6

**Published:** 2023-08-18

**Authors:** Xinlong Dong, Jing-fei Dong, Jianning Zhang

**Affiliations:** 1 Department of Neurosurgery, Beijing Tiantan Hospital, Capital Medical University, No. 119, Nansihuan West Road, Fengtai District, Beijing, China; 2 Beijing Key Laboratory of Central Nervous System Injury, Beijing Neurosurgical Institute, Capital Medical University, Beijing, China; 3 Bloodworks Research Institute, Seattle, WA USA; 4 Division of Hematology, Department of Medicine, School of Medicine, University of Washington, Seattle, WA USA; 5 Department of Neurosurgery, Tianjin Medical University General Hospital, Tianjin, China

**Keywords:** Traumatic brain injury, Extracellular vesicles, Pathological extracellular vesicles, Biological extracellular vesicles, Engineered special purpose extracellular vesicles

## Abstract

Traumatic brain injury (TBI) is a leading cause of injury-related disability and death around the world, but the clinical stratification, diagnosis, and treatment of complex TBI are limited. Due to their unique properties, extracellular vesicles (EVs) are emerging candidates for being biomarkers of traumatic brain injury as well as serving as potential therapeutic targets. However, the effects of different extracellular vesicle subtypes on the pathophysiology of traumatic brain injury are very different, or potentially even opposite. Before extracellular vesicles can be used as targets for TBI therapy, it is necessary to classify different extracellular vesicle subtypes according to their functions to clarify different strategies for EV-based TBI therapy. The purpose of this review is to discuss contradictory effects of different EV subtypes on TBI, and to propose treatment ideas based on different EV subtypes to maximize their benefits for the recovery of TBI patients.

Video Abstract

**Supplementary Information:**

The online version contains supplementary material available at 10.1186/s12964-023-01165-6.

## Introduction

Traumatic brain injury (TBI) is the leading cause of death and disability at all ages, but especially among young people. There are more than 50 million new TBI cases worldwide every year with approximately 1 million deaths. Most survivors, even from mild TBI, will have significantly increased risk of neurodegenerative diseases later in life (e.g., dementia and Parkinson's disease), which will bring serious pain to patients and families and imposing a great public health burden on the society [1]. In recent years, the increasing research on extracellular vesicles has provided new ideas for identifying different TBI types, monitoring the dynamic evolution of the disease, evaluating efficacies of treatments including surgery, and predicting outcomes of the patients [2].

Extracellular vesicles (EVs) are bilayer vesicles secreted by cells or released from injured cells or those undergoing active microvesiculation and they may contain DNA, RNA, intracellular granules, and cytoplasmic proteins of parent cells. Their membrane is enriched in receptors from the transmembrane 4 superfamily, such as CD63、CD9、and CD81 [3] and lipids such as phosphatidylserine, gangliosides, cholesterol, glycosphingolipids and ceramides [4]. These EVs are increasingly recognized as an important class of biological effectors for facilitating intercellular communication, maintaining system homeostasis, and mediating the pathogenesis of neurological diseases, cardiovascular diseases, and cancers [5, 6]. EVs are typically categorized into exosomes, membrane microvesicles, and apoptotic bodies on the basis of their secretion pathway and particle sizes [7–9]. Exosomes measured 30–150 nm in diameter are derived from intraluminal vesicles (ILVs) of multivesicular endosomes (MVEs). Membrane vesicles measure 100–1000 nm in diameter and could be produced by cell budding. Apoptotic bodies are 50-500 nm in diameter and are produced by cells undergoing apoptosis. However, this EV classification has significant limitations. First, the boundaries of this classification are ambiguous, especially as exosomes and microvesicles overlap in size, and current technologies cannot clearly distinguish and identify them solely based on particle size [5]. Second, this classification does consider the structural and functional characteristics of EV subtypes, thus causing confusion in literature reports [10]. Since there are no clear cellular markers and functional characteristics that will clearly separate different types of EVs, traditional markers such as CD9、CD63、CD81、TSG101、Alix、Flotillin-1、HSC70、Actin、MHC I and MHC II are used to identify EV subtypes [9]. A standardized classification of EVs is therefore needed for more comprehensive studies of EVs. In this regard, the MISEV2018 guidelines have proposed the use of the term "extracellular vesicles" and fully explained the size and structure of EVs as well as methods of isolating and identifying them [11]. In this review, we use the term "extracellular vesicles" to include exosomes, membrane vesicles, and apoptotic bodies.

Almost all brain cells can secrete or generate EVs that can cross the blood–brain barrier and enter the circulation [12], so real-time sampling of peripheral blood may offer a convenient means of measuring changes in the brain [2, 13]. Signatures of these EVs can potentially be used to identify the type and severity of TBI evaluation [14], measure clinical efficacies of treatments, evaluate prognosis [2], and predict the risk of long-term sequelae (such as post-traumatic epilepsy, Alzheimer's disease) [13, 15, 16]. More importantly, EVs released into the circulation not only carry the original biological information of the parental cells but also protect their cargo contents, such as nucleic acids and proteins from enzymatic degradation in the blood [17]. They can be transformed into drug carriers for the treatment of TBI and its complications as well [18]. Because of their complexity, the question is whether EVs as a whole are beneficial or detrimental to TBI patients. Answering this question proves challenging for several reasons. First, experimental studies in vitro and in animal models find that different types of EVs can have different or opposite effects on TBI [18, 19]. Second, the same EV subtype may have different effects on various diseases and pathological stages of TBI [20].

This review links the biology of EVs to the pathogenesis of TBI. To more clearly distinguish the subtypes of extracellular vesicles with differential effects on TBI and facilitate the selection of appropriate EV-based therapeutic strategies, we divided EVs into three categories: pathological EVs (PEV), biological EVs (BEV), and drug-loaded engineered special purpose EVs (EEV). PEV mediate the pathophysiological process of secondary damage from TBI, and BEV inhibit the progression of TBI secondary damage and participates in tissue repair and body rehabilitation, and EEV can treat TBI in a targeted and specific manner. This classification does not refer to a specific type of EV, but a collection of many types of EVs with the same function. Furthermore, it is relative, in that specific EVs can be PEV under certain conditions but fall into another class under different circumstances such as different pathological stages of TBI. When EEV are improperly modified to carry drugs to treat TBI patients, unexpected complications may occur, and EEV thus become PEV.

## PEV, BEV and EEV in TBI

### PEV mediate secondary damages from TBI

We summarize the reports on the pathological role of PEV in the current literature (Table 1). It should be pointed out that the difference between membrane vesicles and exosomes highlighted in the early literature not only pertains their size and biogenesis, but also to that the surface of the membrane vesicles is enriched with anionic phospholipid phosphatidylserine (PS). Recent studies have shown that exosome membranes also contain PS [21], but it is not known whether there is a difference in PS contents between exosomes and membrane vesicles. PS is primarily located on the inner membrane of cells, but it becomes exposed on the surface of EVs when the asymmetric distribution of phospholipids is remodeled [8]. However, the biological effects of PS exposed on EVs remains poorly understood except for its procoagulant activity [22]. PS-enriched membrane vesicles and exosomes are closely associated with primary or secondary injury induced by TBI. We have shown that PS exposed on the surface of EVs contributes to the development of consumptive coagulopathy in mice subjected to TBI [23–25]. PS-enriched EVs are therefore collectively considered to be PEV.

**Table 1:** PEV mediates pathological damages after TBI^a^

EV Sources: Cell type/ Tissue /Species	Subset of PEV	Key component of PEV	Animal model/ sampling time point	PEV mediates pathological damages	Mechanisms/Main findings	Ref
Neurons and glial cells/ brain, plasma/Mouse	**BDEV, PS**^**+**^**EV, TF**^**+**^**EV**	**PS and TF on membrane**	**TBI/0.5,1,3 and 6 h post-injury**	**•Coagulopathy** **•Systemic complications**	**•The traumatized brain releases procoagulant BDMPs into the circulation to trigger a disseminated coagulation cascade** **•The abundance of PS and TF on the membrane surface is responsible for the procoagulant activity of BDMP**	**Ye Tian et al.** [23]
Neurons and glial cells/ plasma/ Mouse	**CL**^**+**^** mitochondrion**	**CL on mitochondrial membrane**	**TBI/0.25, 0.5,1,3,7,10,14 days post-injury**	**•Coagulopathy** **•Systemic complications**	**•The mtMP is a major subset of BDEVs** **•Abundant CL on the membrane surface is responsible for mtMPs-triggered coagulation dysfunction after TBI**	**Zilong Zhao et al.** [24]
Neurons and glial cells/ plasma/ Mouse	**BDEV, PS**^**+**^**EV**	**PS on membrane**	**TBI/3,24 h;1,2,3 days post-injury**	**•Coagulopathy** **•Systemic complications**	**•The assembly of Tenase on PS is an important reason for the extrinsic coagulation cascade reaction triggered by BDEV/PS**^**+**^**EV** **•ANV-6L15 prevents the assembly of Tenase on PS to inhibit coagulopathy and systemic complications after TBI**	**Xinlong Dong et al.** [25]
Neurons and glial cells/ plasma/ Mouse	**BDEV, PS**^**+**^**EV**	**PS on membrane**	**TBI/3,6 h;1,3,7 days post-injury**	**•Coagulopathy** **•Systemic complications**	**Lactadherin promotes the clearance of BDEVs by macrophages**	**Yuan Zhou et al.** [26]
Neurons and glial cells/ brain, plasma/ Mouse	**CL**^**+**^** mitochondrion**	**CL on mitochondrial membrane**	**TBI/3 h post-injury**	**Coagulopathy**	**Extracellular mitochondria bind platelets through phospholipid-CD36 interactions and induce α-granule secretion, vesicle formation, and procoagulant activity**	**Zilong Zhao et al.** [27]
Neurons and glial cells/ brain, plasma/ Mouse	**BDEV, PS**^**+**^**EV**	**PS on membrane**	**TBI/3,6 h post-injury, 12 h after BDEV infusion**	**Impair cerebrovascular autoregulation**	**BDEVs cause sudden death in mice by inducing severe vasoconstriction**	**Jiwei Wang et al.** [28]
blood cells /plasma/ Mouse	**Circulating EVs**	**Specific miRNA and chemokines**	**TBI/2, 6, 12 and 24 h post-injury**	**•Dysregulated inflammatory** **•Systemic complications**	**The number of circulating EVs increases after TBI, along with increased numbers of leukocytes in the CNS and liver, exacerbating the acute-phase response**	**Isla Hazelton et al.** [29]
Neurons and glial cells/brain/ Mouse	**BDEV**	**Proinflammatory cytokine IL-1β, inflammasome components and MHCII proteins, etc**	**Stroke/ 1, 3, 7 and 14 days after surgery**	**Dysregulated inflammatory**	**•BDMPs exacerbate neuroinflammation and aggravate ischemic brain injury after stroke** **•Lactadherin exerts anti-inflammatory effects and increases EV clearance, thereby reducing BDEV-induced neurological deficits after stroke**	**Chen Z et al.** [30]
BV2 microglia/culture medium/Mouse	**Microglial-derived EV**	**Pro-inflammatory molecules**	**TBI/24 h post-injury**	**Dysregulated inflammatory**	**EVs loaded with pro-inflammatory molecules can activate microglia after TBI, which may exacerbate neuroinflammatory and systemic immune responses**	**Kumar A et al.** [31]
Brain cells/Brain/Rat	**BDEV**	**EVs-associated miR142**	**TBI/2 weeks post-injury**	**Dysregulated inflammatory**	**EVs-associated miR142 in the cerebral cortex surrounding the traumatic lesion in rats 2 weeks after TBI may further enhance the pro-inflammatory response of activated astrocytes in the region**	**Korotkov A et al.** [32]
PC12 cells/culture medium/Rat	**Neuron-derived EVs**	**miR-21-5p**	**TBI/3 days after administration**	**Dysregulated inflammatory**	**Neuron-derived EVs containing miR-21-5p induced microglial polarization, promoted the release of neuroinflammatory factors and exacerbated neuronal injury**	**Yin Z et al.** [33]
Astrocytes/culture medium/ human	**Astrocytes-derived EVs**	**Specific subset of miRNAs**	**—**	**Dysregulated inflammatory**	**Astrocyte-derived EVs express a specific subset of miRNAs that may play a potential role in modulating inflammatory responses**	**Manoshi Gayen et al.** [34]
Neurons/ brain/ Mouse	**Neurons-derived EV**	**miR-21**	**TBI/1–7 days post- injury**	**Dysregulated inflammatory**	**miR-21 as a potential cargo of neuron-derived EVs may mediate the activation of microglia**	**Harrison EB et al.** [35]
EV in circulating blood /serum /Mouse	**Serum-derived EVs**	**Inflammasome protein**	**TBI/4 and 24 h post-injury**	**•Dysregulated inflammatory** **•Systemic complications**	**•TBI induces EVs containing inflammasome proteins to target the lung and cause acute lung injury** **•Low-molecular-weight heparin blocks EV uptake by recipient cells and thereby inhibits inflammasome activation in the lungs of mice**	**Kerr NA et al.** [36]
Neurons and glial cells, Platelet, Endothelial cells/ plasma/ Mouse	**BDEV, pEVs, eEVs**	**VWF-bound EVs**	**TBI/1,3,4,6,12,24,36,48,72 h post-injury**	**•BBB disruption** **•Coagulopathy** **•Systemic complications**	**•Plasma VWF binds EVs to form VWF-EV complexes, disrupting the integrity of the BBB and increasing its permeability after TBI** **•rADAMTS-13 enhances VWF cleavage to preserve BBB integrity and prevent TBI-induced coagulopathy**	**YingangWu et al.** [37]
Brain endothelial cells/ plasma/Mouse	**eEVs**	**Tight junction** **proteins**	**TBI/24 h post-injury**	**•BBB disruption**	**Brain endothelial cells release eEVs containing TJP and endothelial markers to mediate vascular remodeling after TBI**	**Andrews AM et al.** [38]
Brain endothelial cell/plasma/Rat	**eEVs**	**—**	**Focal inflammatory brain lesions, 2 and 4 h after administration**	**•Dysregulated inflammatory** **•Systemic complications**	**Focal brain injury increased release of EV and initiated an acute-phase response in the liver**	**Couch Y et al.** [39]
Neuroblastoma N2a cells/culture medium/Mouse	**Neuroblastoma-derived EVs**	**Abeta peptides**	**—**	**Neurological disorders associated with TBI**	**EVs carrying Abeta peptides mediate the occurrence of AD**	**Rajendran L et al.** [40]
Neuroblastoma M1C cells/culture medium/ Human	**Neuroblastoma-derived EVs**	**tau protein**	**—**	**Neurological disorders associated with TBI**	**The mechanism by which the majority of tau secreted by M1C cells is released by EVs may explain the unconventional secretion of other aggregation-prone proteins in neurodegenerative diseases**	**Saman S et al.** [41]
SH-SY5Y cells/culture medium/Human	**Neuroblastoma-derived EVs**	**Alpha-synuclein**	**—**	**Neurological disorders associated with TBI**	**Alpha-synuclein released by EVs contributes to the amplification and dissemination of Parkinson's disease-associated pathology**	**Emmanouilidou E et al.** [42]
SH-SY5Y cells were mixed cells expressing TDP-43/Culture medium/Huamn	**EV from cells expressing TDP-43**	**TDP-43**	**—**	**Neurological disorders associated with TBI**	**EVs may contribute to the release of intracellular TDP-43 aggregates to mediate the occurrence of amyotrophic lateral sclerosis**	**Nonaka T et al.** [43]
SH-SY5Y cells/culture medium/Human	**Neuroblastoma-derived EVs**	**Alpha-synuclein**	**—**	**Neurological disorders associated with TBI**	**Alpha-synuclein in EVs aggregates more easily than cytosolic proteins, and aggregated alpha-syn is also released by cells**	**Lee HJ et al.** [44]
Human H4 neuroglioma cells and neurons from mouse/culture medium	**Neuroglioma- and neurons-derived EVs**	**Alpha-synuclein**	**—**	**Neurological disorders associated with TBI**	**Compared with free αsyn oligomers, EV-associated αsyn oligomers were more easily taken up and more toxic to recipient cells**	**Danzer KM et al.** [45]
HEK-293 cells and neurons from mouse/culture medium	**HEK-293 cells- and neurons-derived EVs**	**TDP-43**	**—**	**Neurological disorders associated with TBI**	**Compared with free TDP-43, TDP-43 in EVs was not only preferentially taken up by recipient cells, but also more toxic to recipient cells**	**Feiler MS et al.** [46]
EV in circulating blood /plasma/human	**LEVs and SEV in peripheral circulation**	**Specific mRNA and lncRNA**	**—**	**Neurological disorders associated with TBI**	**Analysis of SEV and LEV cargoes suggests that RNA may serve as novel, readily accessible biomarkers for AD, PD, ALS, and FTD in the future**	**Sproviero D et al.** [47]

^a^*Abbreviations*: *AD* Alzheimer's disease, *ALS* amyotrophic lateral sclerosis, *ANV-6L15: ANV-6L15* fusion protein, *BBB* blood–brain barrier, *BDEV* brain-derived extracellular vesicles, *CL* cardiolipin, *CL*^+^*mitochondrion* CL-enriched mitochondrion, *CNS* central nervous system, *eEVs* endothelial-derived extracellular vesicles, *EVs* extracellular vesicles, *FTD* frontotemporal dementia, *IL-1β* interleukin-1β, *LEVs* large extracellular vesicles, *mtMP* mitochondrial microparticles, *PD* Parkinson's disease, *PEV* Pathological extracellular vesicles, *pEVs* platelet-derived extracellular vesicles, *PS* phosphatidylserine, PS^+^EV PS-enriched extracellular vesicles, *rADAMTS-13* A Disintegrin and Metalloprotease with ThromboSpondin type 1 repeats, member 13, *SEV* small extracellular vesicles, *TBI* Traumatic brain injury, *TDP-43* TAR DNA-binding protein of 43 kDa, *TF* tissue factor, *TF*^+^*EV* TF-enriched extracellular vesicles, *TJP* Tight junction proteins, *VWF* von Willebrand factor

#### PEV and TBI-induced coagulopathy

TBI-induced coagulopathy (TBI-IC) is a common and serious complication of TBI [48, 49], manifested as systemic coagulation disorder and secondary or delayed intracranial or intracerebral hemorrhage, which often results in severe neurological dysfunction and death [25]. The incidence of coagulopathy after TBI is reported to be 32.7–35.2% according to two meta-analyses [50, 51], and most patients with severe TBI have abnormal coagulation tests indicating hypercoagulation [48, 52]. Patients with TBI-IC have a ninefold higher risk of death compared with TBI patients without coagulopathy, leading to a mortality of 35–50% [48, 49, 52]. Despite its high mortality rate, the pathogenesis of TBI-IC remains poorly understood. Our recent studies in mouse models suggest that EVs have multiple roles in triggering TBI-IC [23–25, 37, 53].

**Fig. 1:**
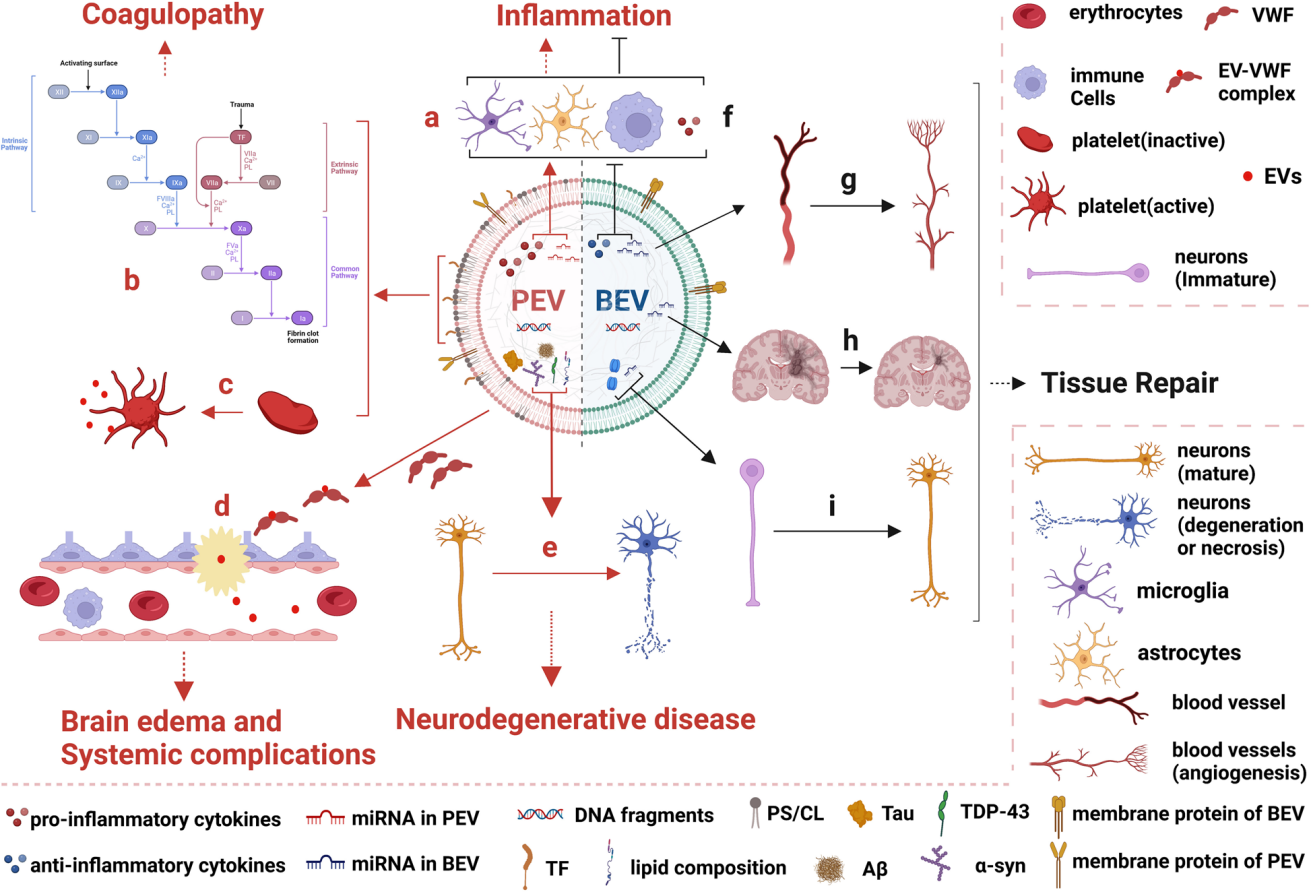
Different effects and therapeutic potential of PEV and BEV on TBI. (**a**) - (**e**): PEV mediate the pathophysiological processes that result in secondary damage from TBI, such as inflammation, coagulopathy, brain edema, systemic complications, and neurodegenerative disease. (**a**) The miRNA and pro-inflammatory cytokines in PEV promote glial and immune cell activation and release of proinflammatory cytokines. (**b**) The PS/CL and TF in PEV trigger and exacerbate the coagulation cascade. (**c**) The PS/CL in PEV activate platelets and causes them to release pEVs. (**d**) The EV-VWF complex disrupts the BBB and EVs enter the peripheral circulation. (**e**) Misfold proteins in PEV cause neuronal degeneration and apoptosis. (**f**) - (**i**): BEV suppress excessive inflammation and participate in tissue repair and regeneration after TBI. (**f**) The miRNAs and anti-inflammatory cytokines in BEV inhibit glial and immune cell activation and release of pro-inflammatory cytokines. (**g**) BEV promote the formation of new blood vessels. (**h**) The miRNA in BEV inhibits hypertrophic glial scar formation. (**i**) The neurotrophins and miRNA in BEV promote neuronal growth and maturation. Abbreviations: Aβ:amyloid β-peptide;α-syn:α-synuclein; BBB: blood–brain barrier; BEV: biological extracellular vesicles; CL: cardiolipin; EVs: extracellular vesicles; PEV: pathological extracellular vesicles; PS: phosphatidylserine; TDP-43: TAR DNA-binding protein of 43 kDa; TF: tissue factor; VWF: von Willebrand factor. Figure created with BioRender.com

We demonstrated that mice subjected to TBI release significant amounts of brain-derived extracellular vesicles (BDEVs) into the circulation, where these BDEVs induce a systemic hypercoagulable state that rapidly develops into consumptive coagulopathy [53]. Key molecules involved in this BDEV-induced systemic hypercoagulation include anionic phospholipids such as PS, which is highly enriched in brain cells [25], and tissue factor (TF) exposed on the membrane surface of BDEVs [23]. The membrane-bound PS and TF allow for the assembly of the tenase complex in the extrinsic coagulation cascade, thus consuming a substantial amount of coagulation factors. In addition, BDEVs, especially extracellular mitochondria (exMT) that are a key component of them [24], can activate platelets and endothelial cells to release platelet-derived EVs (pEVs) and endothelial cell-derived EVs (eEVs) to propagate the intravascular coagulation initiated by BDEVs [25, 27, 37]. These exMTs promote coagulation through the surface exposed anionic phospholipid cardiolipin (CL) [24] and are also metabolically active in generating reactive oxygen species (ROS), which activate platelets through the interaction between the lipid scavenging receptor CD36 on platelets and CL on exMTs [27]. Consistent with our results, Nekludov et al. [54] found that EV counts in cerebral venous blood (regardless of cell origin) were higher in TBI patients than in healthy individuals and that TF-exposed eEVs and P-selectin-exposed pEVs had higher concentrations in cerebral vein samples than in arterial samples. These clinical data further support the notion that PEV mediates the development of coagulopathy after TBI [38, 55–57](Fig. 1b&c).

#### PEV and TBI-induced inflammation

Neuroinflammation is a process of immune activation that mediates the development of secondary cerebral injures during acute TBI [58]. Upon exposure to traumatic injury, damaged meninges, glial cells, and brain parenchyma rapidly release molecules that are collectively termed damage-associated molecular patterns (DAMPs), which release ATP, high-mobility group box protein 1(HMGB1) and other related factors [59–61]. These molecules bind to pathogen-associated molecular patterns (PAMP) and DAMP sensors (such as TLR and purinergic receptors) [62] to assemble inflammasome [63, 64] and activate microglia [65], which produce IL-1b, IL-6, IL-12, TNF-α, metalloproteinases, nitro oxide, and ROS to promote inflammatory responses [66, 67]. As immune cells first to infiltrate the CNS during acute inflammation, neutrophils are recruited to and become activated at the injury site [68], and they propagate the injury-induced local cerebral inflammation through their interaction with microglia and astrocytes [68, 69]. Monocytes and T cells are then recruited to the damaged area, where monocytes are transformed into macrophages to clean up debris and damaged cells [70] and T cells produce neuroprotective cytokines involved in neuroinflammation [71]. The TBI-induced neuroinflammation can either subside over time or become a persistent chronic inflammatory state [72]. While neuroinflammation is critical for debris clearance, tissue repair, and nerve regeneration after TBI, dysregulated inflammation can lead to additional acute and chronic damages to the brain [58].

Several lines of evidence suggest that PEV contribute to dysregulated inflammation associated with TBI (Fig. 1a). First, PEV act as a mediator for the development of excessive or persistent inflammation in TBI [19]. The levels of circulating EVs in mice subjected to TBI are significantly increased, and these EVs exacerbate and propagate the inflammatory response after TBI [29], whereas neuroinflammation is effectively suppressed and the neurological function is significantly improved with the removal of plasma EVs [30]. Second, PEV are reported to regulate glial cells to propagate and amplify the inflammatory response after TBI by delivering a large number of pro-inflammatory mediators and specific miRNAs [31–33]. As the first responder and a major player in TBI-induced inflammation, microglia (similar to macrophages) are traditionally divided into a pro-inflammatory M1-like phenotype and an anti-inflammatory M2-like phenotype [58, 73], even though new classifications based on RNA-sequencing at the single-cell level are increasingly recognized for establishing a clear map of microglia and macrophages at different stages of TBI [72]. We recognize that the term M1 and M2 microphages are also called pro-inflammatory (M1) and pro-regenerative (M2) glia cells and macrophages in recent report. To avoid confusion, both terms are included in this review. The neuron-derived PEV carrying microRNA-21-5p induce pro-inflammatory microglia (M1 microglia) to exacerbate neuroinflammatory cytokine release, inhibit neurite regeneration, and promote neuronal apoptosis, thus causing a cyclic cumulative damage [33]. Furthermore, more EV-associated miR142 exists in the cerebral cortex surrounding the traumatic lesion in rats 2 weeks after TBI and may further enhance the pro-inflammatory response of activated astrocytes in the region [32]. There are three possible mechanisms through which EVs affect target cells. First, membrane EVs directly fuse with the membrane of target cells or with the endosomal membrane if EVs are endocytosed to release their miRNAs into target cells [74], or alternatively EV-carried microRNA species bind to target mRNAs to reduce their translation [75]. Second, miRNAs carried by EVs bind to pattern recognition receptors in the endosomal compartment, such as Toll-like receptors 7/8 (TLR7/8) [76], to trigger pro-inflammatory responses [77]. Third, neuron- or glial cell-derived PEV directly participate in central nervous system (CNS) inflammatory responses and exacerbate secondary damage after TBI. Kumar et al. [31] found that PEV released by microglia after TBI are rich in the proinflammatory mediators IL-1β and miR155 and further propagate the inflammatory response within the cerebral cortex of mice subjected to severe TBI. EVs released from primary human astrocytes activated by IL-1 express a specific subset of miRNAs [34], in which MiR-30d upregulates pro-inflammatory cytokines including IL-1 to promote autophagy and apoptosis in these cells [78]. Similarly, Harrison et al. [35] found that miR-21-enriched EVs were pro-inflammatory and induced neuronal necroptosis in mouse models of TBI. The signaling pathways and molecular mechanisms of PEV carrying different miRNAs and pro-inflammatory mediators directly involved in the inflammatory response after TBI remains to be further studied. Finally, PEV may mediate inflammation crosstalk between CNS and systemic organs. In other words, PEV-mediated inflammatory injury after TBI involves both circulating PEV crossing the damaged blood–brain barrier (BBB) and exacerbating CNS inflammation and injury [29], and CNS-derived PEV crossing the damaged BBB into the peripheral circulation, resulting in acute organ damage [36].

#### PEV and brain edema after TBI

Cerebral edema during the acute state of TBI can increase intracranial pressure, resulting in secondary ischemic cerebral tissue injuries, brain herniation, and death [79, 80]. The disruption of the BBB is the most common cause of vasogenic edema [80]. In addition to traumatic injury, secondary neuroinflammation and oxidative stress further damage the BBB, significantly increasing its permeability and perivascular fluid accumulation [81]. The permeability of BBB increases through two interconnected processes: increasing paracellular transport and causing transcytosis across endothelial cells. For the former, mechanical injury, neuroinflammation, and oxidative stress disrupt the tight junction structure between endothelial cells, leaking normally inadmissible components into the extravascular space, such as immune cells that intensify the local inflammatory reaction to propagate BBB damage in a vicious circle [79, 82, 83]. For the latter, the number of endothelial cell caveolae increases rapidly shortly after TBI to allow the diffusion of proteins across endothelial cells via liquid-phase transcytosis and transendothelial channels, leading to transport and accumulation of macromolecules and serum proteins in the interstitial space of the brain [79, 82, 83].

We have shown in mouse models that PEV enhance BBB permeability to promote cerebral edema and a systemic hypercoagulable state during the acute phase of TBI [25, 37] (Fig. 1d). BDEVs released by injured brains also stimulate endothelial cells to secrete the hyperadhesive von Willebrand factor (VWF), which activates platelets to generate procoagulant and proinflammatory pEVs in fluid phase. These VWF-bound EVs adhere to endothelial cells of the BBB through the interaction with CD62p [84] and integrin αvβ3 [85] to activate endothelial cells and generate procoagulant eEVs [37]. Reducing the hyperadhesive activity of VWF by enhancing VWF proteolysis or blocking its active site prevented EV-induced endothelial injury, coagulopathy, and neurological deficits associated with severe TBI [26, 37, 86]. Consistent with our results, Andrews et al. found that the brain endothelial cells of TBI mice release eEVs containing claudin and endothelial markers to increase BBB permeability [38]. Because of the high heterogeneity of PEV from different types of cells, efforts are needed to differentially identify specific components responsible for causing BBB permeability, neuroinflammation, oxidative stress, and coagulopathy and their underlying mechanisms.

#### PEV and systemic complications after TBI

Systemic complications of TBI are common and contribute to the high mortality of patients [1]. These complications involve the lungs, heart, coagulation system, kidneys, and liver [87, 88], but how a relatively localized injury to the brain is disseminated systemically remains poorly understood. Several factors may collectively contribute to the systemic effects of TBI. The first is the "catecholamine surge”, which refers to the massive release of epinephrine and norepinephrine from the hypothalamic-pituitary axis during acute TBI, resulting in the constriction of peripheral blood vessels [89]. The second is TBI-induced inflammation. The third is PEV-induced systemic inflammation, immune dysregulation, and intravascular coagulation. The lungs are the most common organ that develops secondary injury post TBI [87, 88], usually manifesting as acute lung injury, acute respiratory distress syndrome, pneumonia, pleural effusion, pulmonary edema, and pulmonary thromboembolism [25, 90]. Kerr et al. found that EVs carrying proinflammatory cytokines were released into the peripheral circulation after TBI in experimental mice, and these EVs were endocytosed by pulmonary cells including endothelial cells to trigger inflammasome activation and resultant lung injury [36, 91]. Hazelton and Couch et al. reported that PEV serve as communication mediators between the nervous system and liver, to trigger systemic inflammation and exacerbate injuries to the nervous system and the liver during acute TBI [29, 39]. PEV are also the key mediator of TBI-IC and trigger secondary injuries to other organs [23, 25, 26].

#### PEV and neurological disorders associated with TBI

Increasing evidence supports TBI as a major risk factor for long-term neurological diseases, especially neurodegenerative diseases such as Alzheimer's and Parkinson's disease, further strengthening the argument that acute TBI can evolve into chronic diseases [1, 92]. A meta-analysis of samples from 4,639 patients by Fleminger et al. [93] found that a history of TBI was associated with a 2–fourfold increased risk of Alzheimer's disease (AD) late in life and that the more severe the injury, the higher the risk for AD will be. Similarly, repeated TBI after age 55 increases the risk of Parkinson's disease (PD) by 44% over the following 5–7 years, and that risk is positively associated with the severity of TBI [94]. However, we would like to point out that, while TBI as a long term risk for neurodegenerative disease has been extensively studied in clinical settings and in animal models, the vast majority of these studies have been conducted on patients with mild to moderate TBI, with very limited information regarding the risk of patients with severe TBI for neurodegenerative diseases [95]. In animal studies, the long-term effects on cognitive function have also been investigated with mice or rats exposed to mild to moderate TBI. Findings from limited reports on severe TBI patients are not consistent. For example, in a study of the working-age population, a history of moderate-to-severe TBI is associated with an increased risk for future dementia but not for Parkinson disease or amyotrophic lateral sclerosis [96]. In contrast, a study of pooled clinical and neuropathology data from three prospective cohort studies shows that TBI with loss of conciseness (TBI severity was not defined by common measurements such as GCS or ISS in this study) has increased risks for Lewy body accumulation, progression of Parkinsonism, and Parkinson's disease, but not dementia, Alzheimer’s disease, neuritic plaques, or neurofibrillary tangle [94]. More importantly, we were unable to find any studies in the literature that have evaluated the effects of TBI treatments (e.g., decompressive craniectomy) on the development of neurodegenerative diseases. Since surgery and other TBI resuscitation measures can be significant confounding variables for the long-term outcomes of patients, it proves very challenging to accurately estimate risk for neurodegenerative diseases in patients with severe TBI, who will undergo extensive surgical and non-surgical treatments.

The typical pathology of TBI-associated AD is similar to that of other causes, i.e., amyloid β-peptide (Aβ) aggregates into extracellular amyloid plaques and hyperphosphorylated tau accumulates intracellularly to form neurofibrillary tangles [97], and Lewy bodies (LBs) and Lewy neurites (LNs) in PD contain oligomerized α-synuclein (α-syn) [98].

Evidence also shows that PEV play an important role in developing TBI-associated neurodegenerative diseases [99] (Fig. 1e). EV biogenesis is an important complementary pathway for clearance of misfolded proteins, especially when lysosomal function is compromised [100]. When lysosomes are impaired in their ability to remove toxic proteins, cells initiate or upregulate EV biogenesis to achieve the same effect as the intracellular degradation of harmful components by secreting EVs containing toxic proteins [101]. Furthermore, EVs carry pathogenic protein aggregates and are able to spread neurodegeneration-associated protein aggregates throughout the brain [100], such as Aβ [40] and tau in AD [41, 102], α-synuclein in PD [42, 44], TAR DNA-binding protein of 43 kDa (TDP-43) in amyotrophic lateral sclerosis [43], and huntingtin protein in Huntington's disease [103]. Finally, cells have a higher rate of endocytosing misfolded proteins packed in EVs than free misfolded proteins. As such, EVs carrying misfolded proteins are likely to be more toxic to neurons [41, 45, 46]. In addition, RNAs packed in EVs also contribute to the development of neurodegenerative diseases after TBI [47]. For example, miRNA-9, miRNA-29a and b, and miRNA-146a in blood and cerebrospinal fluid are involved in the formation of misfolded proteins and related inflammatory processes in AD [104–106]. Estes et al. reported that the lipid component of EVs plays an important role in the progression of neurodegeneration [100] by promoting the aggregation and spread of pathogenic protein aggregates. In conclusion, increasing evidence supports the involvement of PEV in the development of chronic neurological disease long after TBI, but their specific activities remains to be further defined.

### Protective and healing effects of BEV in TBI

In addition to their detrimental effects, EVs may also have protective or healing effects and can call beneficial EVs (i.e., BEV) derived from either different classes of EVs or differential components of the same types of EVs (Table 2). Efforts to identify, characterize, and separate detrimental from beneficial EVs have been ongoing but face significant challenges to overcome. Apart from their intrinsic activities, the same types of EVs can be both detrimental and beneficial depending on their targets, environments, and times of their actions.

**Table 2:** Protective and healing effects or potential therapeutic value of BEV on TBI^a^

**EV Sources: Cell type/ Tissue/species**	**Subset of BEV**	**Key component of BEV**	**Animal model/** S**ampling time point**	**Protective and healing effects of BEV**	**Mechanisms/Main findings**	**Ref**
Stem cells from human exfoliated deciduous teeth (SHED)/culture medium/human	**SHED-derived EVs**	**miR-330-5p**	**TBI/48 h after treatments, within 21 days after treatments**	**•Anti-inflammatory** **•Improve neurological function**	**SHED-EVs carrying miR-330-5p inhibited the secretion of inflammatory cytokines and promoted the recovery of motor function in TBI rats**	**Li Y et al.** [107]
Astrocytes/culture medium/mouse	**Astrocytes derived EVs**	**miR-873a-5p**	**TBI/ 1, 3, 7 and 14 days post-injury**	**•Anti-inflammatory** **•Improve neurological function**	**Astrocyte-derived EVs carrying miR-873a-5p inhibited the NF-κB signaling pathway, thereby attenuating microglia-mediated neuroinflammation and improving neurological deficits after TBI**	**Long X et al.** [108]
BV2 microglial cells/ culture medium/mouse	**Microglia-derived EVs**	**miR-124-3p**	**TBI/3, 7, 14** **, 21, 28,32 and 35 days post-injury**	**•Anti-inflammatory** **•Neuroreparative functions**	**Microglia-derived EVs carrying miR-124-3p can suppress neuronal inflammation and promote neurite outgrowth after TBI**	**Huang S et al.** [109]
Astrocytes/culture medium/Rat	**Astrocytes-derived EVs**	**Specific** **subset of miRNAs**	**—**	**Neuroprotective function**	**•Astrocytes modify miRNAs in EVs in response to changes in the extracellular microenvironment** **•Modified miRNAs regulate synaptic stability and neuronal excitability to reduce the activity of target neurons**	**Chaudhuri AD et al.** [110]
Neuron/cerebral cortex/Rat	**Neuron-derived EVs**	**miR-181c-3p**	**Ischemic brain injury/1–5 days after surgery**	**•Anti-inflammatory**	**Cortical neuron-derived EVs carrying miR-181c-3p downregulate CXCL1-associated neuroinflammation and thus exert protective effect on IBI rats**	**Song H et al.** [111]
Neutrophils /plasma/human	**Neutrophils-derived EVs**	**PS on membrane**	**—**	**Anti-inflammatory**	**Neutrophils release potent anti-inflammatory factors in the form of EVs at the earliest stages of inflammation and provide the impetus for resolution of inflammation**	**Gasser O et al.** [112]
Mesenchymal stem cells /culture medium/Rat	**MSC-EV**	**—**	**TBI/1, 4, 7, 14, 21, 28, and 35 days post-injury**	**•Anti-inflammatory** **•Neuroreparative functions** **•Improve neurological function**	**MSC-derived EVs promote endogenous angiogenesis and neurogenesis and reduce inflammation after TBI are important reasons for functional recovery of TBI rats**	**Zhang Y et al.** [113]
Mesenchymal stem cells /culture medium/Rat	**MSC-EV**	**miR-133b**	**Stroke/1, 3, 7, 14 days after surgery**	**•Neuroreparative functions** **•Improve neurological function**	**MSCs-derived EVs transfer miR-133b to astrocytes and neurons, promoting neurite remodeling and functional recovery after stroke**	**Xin H et al.** [114]
Astrocyte/brain/Mouse	**Astrocyte-EV**	**Synapsin**	**—**	**•Neuroreparative functions** **•Neuroprotective function**	**Under conditions of high neuronal activity and/or oxidative stress, synapsin released by glial cell-derived EVs promote neurite outgrowth and neuronal survival by modulating the interaction between glial cells and neurons**	**Wang S et al.** [115]
Astrocytes/culture medium /Mouse	**Astrocytes-EV**	**GJA1-20 k**	**TBI/6 days after treatments**	**Neuroprotective function**	**Compared with the GJA1-20 k-knockout EV control group, GJA1-20 k-carrying EVs were taken up by neurons and downregulated the apoptosis rate and upregulated the mitochondrial function to promote neuronal recovery**	**Chen W et al.** [116]
Schwann cells/culture medium /Rat	**Schwann cells-EV**	**p75-Neurotrophin Receptor**	**Sciatic nerve injury/1–5 days post-injury**	**Neuroreparative functions**	**SC-derived EVs significantly enhanced axonal regeneration in vitro and promoted repair of injured sciatic nerves in vivo**	**Lopez-Verrilli MA et al.** [117]
Schwann cell/culture medium /Rat	**rSC-EV**	**miRNA-21**	**—**	**Neuroreparative functions**	**The expression of miRNA-21 is responsible for the pro-regenerative ability of rSC-EVs, which is associated with PTEN downregulation and PI3 kinase activation in neurons**	**Lopez-Leal R et al.** [118]
Adipose-derived stem cells/culture medium /Rat	**ADSC-EV**	**miRNA-26b**	**Sciatic nerve injury/ 8 weeks after treatment**	**Neuroreparative functions**	**miRNA-26b in ADSC-EVs moderately reduces autophagy of damaged SCs by downregulating Kpna2, thereby promoting remyelination**	**Yin G et al.** [119]
Adipose stem cell/culture medium/Rat	**ADSC-EV**	**—**	**—**	**Neuroreparative functions**	**The proliferation of SCs was significantly enhanced after ingesting ADSC-EVs, which may be an important mechanism for ADSC-EVs to promote sciatic nerve repair**	**Haertinger M et al.** [120]
Human umbilical vein endothelial cell-derived cell line EA.hy926 and human lung fibroblasts /culture medium /Human	**HucMSC-EV**	**Wnt4**	**Rat skin burn model/ 1 week and 2 weeks after treatment**	**Cutaneous wound healing**	**hucMSC-EV-mediated Wnt4 induces β-catenin activation in endothelial cells and promotes angiogenesis, which may be an important mechanism of cutaneous wound healing**	**Zhang B et al.** [121]
Human umbilical cord mesenchymal stem cells/culture medium /Human	**HucMSC derived EV**	**—**	**Spinal cord injury/1 and 8 weeks after injury**	**•Anti-inflammatory,** **•Improve neurological function**	**hucMSC-derived EVs reduce inflammation to promote healing of the injured spinal cord**	**Sun G et al.** [122]
Human umbilical cord mesenchymal stem cells/culture medium /Human	**HUCMSC‐EVs**	**—**	**Sciatic nerve injury/2, 4,** **6 and 8 weeks after injury**	**•Anti-inflammatory** **•Neuroreparative functions,** **•Improve neurological function**	**HUCMSC-EVs provide a favorable microenvironment for nerve regeneration to promote functional recovery and nerve regeneration**	**Ma Y et al.** [123]
Bone Mesenchymal Stem Cells/culture medium /Rat	**BMSC-Derived EV**	**—**	**TBI/ 1, 3, 7, and 14 days post-injury**	**•Anti-inflammatory,** **•Improve neurological function**	**BMSCs-EVs regulate the polarization of microglia/macrophages to suppress early neuroinflammation in TBI mice, thereby exerting a neuroprotective effect**	**Ni H et al.** [124]
Mouse and human bone marrow, gingival, and skin MSCs/culture medium /mouse and human	**MSC-derived EV**	**Interleukin-1 receptor antagonist**	**Cutaneous wound/3,5, 7, 10, and 14 days after wound creation**	**Cutaneous wound healing**	**MSCs produce and release sEVs-associated interleukin-1 receptor antagonists to promote gingival wound healing through the Fas/Fap-1/Cav-1 cascade**	**Kou X et al.** [125]
Microglia/culture medium/mouse	**Microglia-EVs**	**miR-5121**	**TBI/1 and 3 days post-injury**	**•Neuroreparative functions** **•Improve neurological function**	**•Overexpression of miR-5121 in EVs improves motor function of TBI mice** **•miR-5121 may directly target RGMa to promote neurite outgrowth and synaptic recovery**	**Zhao C et al.** [126]
Mesenchymal stem cells /culture medium/Rat	**MSC-Derived miR-133b EV**	**miR-133b**	**Spinal Cord Injury/ 12, 24 h, 2, 3, 4, 5, 7,9 and 14 days post-injury**	**•Neuroprotective function** **•Neuroreparative functions** **•Improve neurological function**	**EVs loaded with miR-133b protect neurons and promote axonal regeneration and recovery of hindlimb motor function in SCI rats**	**Li D et al.** [127]
Mesenchymal stem cells/culture medium /Human	**MSCs-EV**	**CD63 and CD81 on membrane**	**TBI/6, 12 h, 28–33 and 35 days post-injury**	**•Anti-inflammatory** **•Improve neurological function**	**CD63**^**+**^**CD81**^**+**^**EVs isolated from mesenchymal stromal cells rescue cognitive impairment after TBI**	**Kim DK et al.** [128]
Bone marrow mesenchymal stem cells/culture medium/ Rat	**BM-MSCs EV**	**—**	**Diabetes/1–5 days after treatment**	**Improve neurological function**	**Bone marrow-derived mesenchymal stem cells transfer EVs to damaged neurons and astrocytes to improve diabetes-induced cognitive impairment**	**Nakano M et al.** [129]
Bone marrow mesenchymal stem cells/culture medium/ Rat	**BM-MSC-Derived miR-124 EV**	**miR-124**	**TBI/3, 7, 14, 21, and 28 days post-injury**	**•Anti-inflammatory** **•Neuroreparative functions** **•Improve neurological function**	**EVs carrying miR-124 promote M2 polarization of microglia and improve hippocampal neurogenesis and functional recovery of TBI rats**	**Yang Y et al.** [130]
Mesenchymal Stromal Cells/Culture medium/ Rat	**MSCs-EV**	**Specific miRNA、messenger RNAs and proteins, etc**	**Stroke/ 1,** **3, 7, 14, 21, and 28 days after surgery**	**•Neuroreparative functions** **•Improve neurological function**	**MSC-EVs enhanced neurite remodeling, neurogenesis, and angiogenesis and improved functional recovery in stroke rats**	**Xin H et al.** [131]
Bone marrow-derived mesenchymal stem cells/Culture medium/Human	**MSCs-EV**	**—**	**Status epilepticus/ 24 h after** s**tatus epilepticus**	**•Anti-inflammatory** **•Neuroreparative functions,** **•Improve neurological function**	**MSC-derived A1-EVs attenuate inflammation and prevent abnormal neurogenesis and memory dysfunction after status epilepticus**	**Long Q et al.** [132]
Bone marrow mesenchymal stem cells /Culture medium/Human	**MSCs-EV**	**—**	**Autism/3 weeks after treatment**	**Improve neurological function**	**Mesenchymal stem cell-derived EVs improve autism-like behavior by intranasal administration in BTBR mice**	**Perets N et al.** [133]
Umbilical cord mesenchymal stem cells /Culture medium /Human	**UCMSCs-EV**	**Vascular endothelial growth factor C, angiopoietin-2, and fibroblast growth factor-2, etc**	**Nerve injury-induced pain/within 18 days after surgery**	**•Anti-inflammatory** **•Neuroreparative functions** **•Improve neurological function**	**Umbilical cord MSC-EVs inhibit spinal nerve ligation-induced neuroinflammation and promote the expression of anti-inflammatory cytokines and neurotrophic factors, and may be candidates for the treatment of pain caused by nerve injury**	**Shiue SJ et al.** [134]
Umbilical cord mesenchymal stem cells/Culture medium /Human	**UCMSCs-EV**	**Specific protein and functional RNAs, etc**	**Nerve injury-induced pain/within 21 days after surgery**	**•Anti-inflammatory** **•Neuroreparative functions** **•Improve neurological function**	**UCMSCs-EVs exert analgesic, anti-inflammatory, and neurotrophic effects in a spinal nerve ligation-induced pain model**	**Hsu JM et al.** [135]
Mesenchymal stem cells/Culture medium /Human	**MSCs– EV**	**—**	**TBI/ 1, 4,7, 14, 21, 28 and 31–35 days post-injury**	**•Anti-inflammatory** **•Neuroreparative functions,** **•Improve neurological function**	**MSCs–EVs not only reduced neuroinflammation and hippocampal neuron loss, but also promoted angiogenesis and neurogenesis, significantly improving sensorimotor and cognitive functions in TBI rats**	**Zhang Y et al.** [136]
Mesenchymal stem cells/Culture medium /Human	**MSCs– EV**	**—**	**TBI and hemorrhagic** **Shock/1–7 days post injury**	**•Anti-inflammatory** **•Neuroreparative functions,** **•Improve neurological function**	**In a large animal model of TBI and hemorrhagic shock, early single-dose MSCs–EV treatment attenuated nerve damage by suppressing inflammation and apoptosis, and promoted neuroplasticity within 7 days**	**Williams AM et al.** [137]
ESC-derived mesenchymal stem cell /Culture medium /Human	**MSCs– EV**	**Functional proteins and RNA**	**Heart model of ischemia/24 h after reperfusion**	**Tissue repair**	**MSCs–EV reduced infarct size in a mouse model of myocardial ischemia/reperfusion injury**	**Lai RC et al.** [138]
Multipotent human bone marrow derived mesenchymal stem cells/Culture medium /Human	**hMSC-EV**	**—**	**TBI/1, 4, 7, 14, 21, 28 and 33–35 days post-injury**	**•Anti-inflammatory** **•Neuroreparative functions,** **•Improve neurological function**	**hMSC-EV significantly improved functional recovery of TBI rats by promoting endogenous angiogenesis and neurogenesis and reducing neuroinflammation**	**Zhang Y et al.** [139]
Mesenchymal stem cells/Culture medium /Human	**MSCs-EV**	**—**	**Skin graft/within 15 days after transplantation**	**•Anti-inflammatory** **•Tissue repair**	**MSCs-EV are immunocompetent and enhance mouse skin allograft survival**	**Zhang B et al.** [140]
Bone marrow-derived mesenchymal stem cells/Culture medium/Rat	**BDNF-induced MSCs-EV**	**miR-216a-5p**	**TBI/1, 7, 14, 28 and 31–35 days post-injury**	**•Anti-inflammatory** **•Neuroreparative functions,** **•Improve neurological function**	**Compared with MSCs-EV, BDNF-mediated MSCs-EVs better promote neurogenesis and inhibit apoptosis after TBI in rats, and the mechanism may be related to the high expression of miR-216a-5p**	**Xu H et al.** [141]
Mesenchymal stem cells/Culture medium /Human and Mouse	**MSCs-EV**	**Specific miRNA**	**Hypoxia-induced pulmonary hypertension/ 2, 4, 7 and 11 days in hypoxia, 3 weeks of hypoxic exposure**	**Protect the lungs**	**MSCs-EV inhibited the hyperproliferative pathway to suppress pulmonary hypertension and exerted pleiotropic protective effects on the lung**	**Lee C et al.** [142]
Bone marrow-derived mesenchymal stem cells/Culture medium /Mouse	**EV from MSCs of ischemic Preconditioning**	**miR-22**	**Myocardial infarction/4 weeks after treatment**	**Cardioprotective function**	**miR-22 in MSCs-EV after ischemic preconditioning targets Mecp2 for cardioprotection**	**Feng Y et al.** [143]

^a^*Abbreviations ADSC* adipose-derived stem cells, *BDNF* brain-derived neurotrophic factor, *BEV* Biological extracellular vesicles, *BM-MSCs* Bone marrow mesenchymal stem cells, *BMSC* Bone Mesenchymal Stem Cells, *CD63*^+^*CD81*^+^*EVs CD63*^+^ *and CD81*^+^-enriched extracellular vesicles, *EV* extracellular vesicles, *GJA1* gap junction alpha 1, *HucMSC* human umbilical cord mesenchymal stem cells, *IBI* ischemic brain injury, *MSC* mesenchymal stromal cells, *PS* phosphatidylserine, *rSC* repair Schwann cells, *SC* Schwann cells, *SCI* spinal cord injury, *TBI* traumatic brain injury, *UCMSCs* umbilical cord mesenchymal stem cells

#### BEV and excessive inflammation after TBI

TBI-induced neuroinflammation plays a key role in repairing disrupted BBB, clearing cellular debris, and releasing trophic factors, but its dysregulation could exacerbate damages to the nervous system, slow the process of tissue repair, and promote the transition to a chronic inflammatory state [72]. Because of these paradoxical post-TBI inflammatory responses, attempts to suppress the inflammatory response have not only failed to improve clinical outcomes for patients during the acute phase of TBI [144, 145] but may increase mortality [146]. The paradoxical role of post-TBI inflammatory responses is also reflected in the function of EVs. EVs released from injured brains are involved in both pathological processes to aggravate nervous system damage as well as the process of tissue repair and healing.

BEV could inhibit the development of excessive inflammation after TBI (Fig. 1f). Notably, microglia-mediated inflammation-associated EVs may be the focus of research to suppress TBI dysregulated inflammation [31]. EVs can stimulate the transition of microglia from pro-inflammatory to pro-regenerative (M1 to M2 transition) [107]. For example, EVs derived from activated astrocytes carrying miR-873a-5p can serve as BEV to mediate the communication between astrocytes and microglia, inhibiting the NF-κB signaling pathway to reduce microglia-mediated neuroinflammation and improve neurological function in TBI mice [108]. Microglia-derived EVs carrying miR-124-3p may also play an anti-inflammatory role by targeting the PDE4B gene to inhibit the activity of the mTOR signaling pathway, thus suppressing neuroinflammation and promoting neurite outgrowth [109]. Astrocytes, the most abundant glial cells in the human brain, modulate neuronal excitability to alter their EV composition to suppress inflammation [20, 110]. EVs released from cortical neurons were protective against ischemic injury to the brain in rats, as they contain miR-181c-3p that reduces the expression of CXCL1 and the production of inflammatory cytokines in astrocytes to suppress excessive inflammation. It should be noted that this study used a rat model of ischemic brain injury and not TBI, but ischemia is a major contributor to the secondary injuries of TBI [111]. Interestingly, neutrophils release potent anti-inflammatory factors carried by their EVs at the earliest stages of inflammation. Although counterintuitive, these EVs increase the release of transforming growth factor β1 (TGFβ1), the externalization of PS, and the downregulation of human macrophage activity to suppress early hyperinflammatory responses [112]. These reports suggest that the distinction between PEV and BEV may not necessarily exist in their parental cells or in the pathological stage of TBI. However, the complexity and overlap of the "damaging effect" and "protective effect" of neuroinflammation after TBI hinder the development of effective strategies for overcoming detrimental effects of EVs while preserving their beneficial effects [58].

#### BEV and tissue repair after TBI

BEV can target receptor cells to participate in the repair and regeneration of neural tissue (Fig. 1g&i). For example, EVs derived from mesenchymal stromal cells (MSCs) significantly increase the number of newly formed neurons and endothelial cells in the dentate gyrus of TBI rats, thereby promoting functional recovery and neurovascular remodeling [113]. These MSC-derived EVs also deliver miR-133b to astrocytes to down-regulate the expression of connective tissue growth factor (CTGF), reduce the formation of scar tissues (Fig. 1h), and promote functional recovery in animal models of ischemic stroke [114]. Astrocyte- and microglia-derived EVs can modulate the interaction between glia and neurons to promote neurite outgrowth and neuronal survival, the mechanism that is closely related to their enrichment of neuroprotective and neurotrophic factors, such as apolipoprotein and synapsin [20, 109, 115]. Consistent with these observations, Chen et al. [116] found that the gap junction alpha 1 -20 kDa (GJA1-20 k) in astrocyte-derived EVs attenuates the phosphorylation of connexin 43 (CX43) to protect mitochondrial function and reduce cell death, thereby protecting and repairing injured neurons in TBI rats.

Different from the CNS, peripheral nerves with stronger regenerative capacity can better reflect the important role played by BEV in tissue repair [117, 147]. Lopez-Leal et al. [118] show that the pro-regenerative capacity of Schwann cell-derived EVs is attributed to increased expression of miRNA-21, which downregulates PTEN (a major negative regulator of neuronal regeneration) and PI3-kinase activation to promote axonal regeneration in neurons. Multiple studies have shown that miRNAs in MSC-derived EVs mediate the expression of Schwann cell activating genes to promote the proliferation of Schwann cells and improve remyelination [119, 120, 148]. In addition, MSC-derived EVs also act as a key regulator of angiogenesis to increase the number of endothelial cells and the formation of new blood vessels [113, 121] as well as suppressing excessive inflammation [122–125].

#### BEV and recovery of neurological function after TBI

The neural function recovery from TBI-induced injury is a multi-step process [1] in which BEV play a critical role [108]. First, motor coordination injured by TBI has been shown to be significantly improved in TBI mice treated with EVs overexpressing miR-5121 [126]. Furthermore, spinal cord injury induced in rats can be repaired by miR-133b carried by MSC-derived EVs through the activation of the ERK1/2, STAT3, and CREB-participating pathways and the inhibition of RhoA expression [127]. BEV can also improve sensory, cognitive, and learning functions [113, 128, 129] by, at least in part, improving hippocampal function after brain injury [130]. In addition to TBI, BEV have also been reported to improve neurological function in models of stroke [131], status epilepticus [132], autistic behavior [133], and peripheral nerve injury [134, 135]. However, more studies are needed to clarify which parts of the brain repaired by BEV lead to these neurological improvements. Moreover, it appears more promising to research means of manipulating EVs into driving the immune reaction in a direction that favors wound repair and functional recovery, instead of completely eliminating neuroinflammation after TBI, as a new pathway for improving outcomes of patients with TBI. One such approach is to use EVs as a vehicle for targeted delivery of therapeutic or regulatory agents.

### EEV as a drug carrier to treat TBI in a targeted manner

The unique physicochemical properties of EVs make them an ideal drug carriers because they offer several distinct advantages. First, they can be readily made from parental cells or synthetic materials and are immune-tolerant and easy to store [7, 149, 150]. Second, they can be selectively packed with DNA, RNA, protein, lipid and small molecule drugs that are delivered to targeted cells [17, 151]. Third, the lipid bilayer ensures that these membrane EVs are resistant to enzymatic digestion in the blood and thus ensure sufficient delivery of their cargo loads [17]. Finally, their small sizes allow them to pass through the BBB to the brain parenchyma [150]. For these reasons, research on EEV drug-loaded therapy has increased exponentially, especially in relation to cancer therapies, wound healing, and cardiac remodeling [17, 152–154].

At present, there are two main sources of EEV: directly modifying natural EVs and imitating EVs to produce biomimetic EVs [155]. Current research on EEV in the field of TBI is far less than that of cancer or other areas. For example, EVs loaded with curcumin or a signal transducer and activator of transcription 3 (Stat3) inhibitor induce microglial apoptosis and suppress brain tumor growth [156]. Modified EVs and siRNA together promote the transformation of microglia and macrophages from pro-inflammatory to pro-regenerative (M1-M2 transition) as well as reduction of inflammatory responses and neuronal damage, thereby promoting functional recovery in spinal cord injury in mice [157]. We will discuss potential therapeutic uses of EEV for TBI by referring to recent reports of EEV usage in cancer or other research fields.

#### Modified EVs

**Fig. 2:**
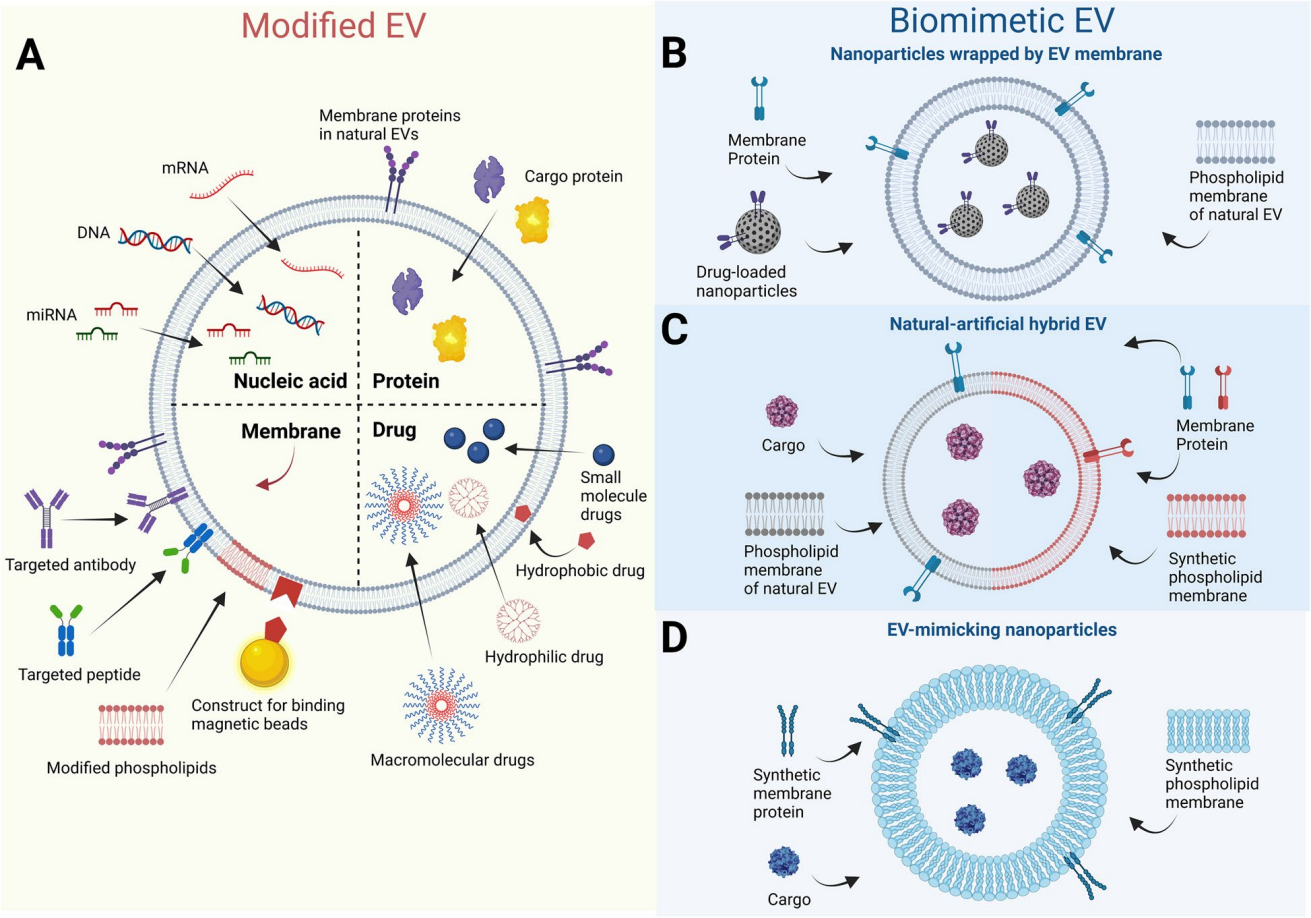
Designs and main types of EEV. (**A**) Modified EV design strategies: Nucleic acids, proteins, and drugs are loaded into EV cargo, or antibodies, peptides, phospholipids, and special materials are used to modify the EV membrane. (**B**) - (**D**) Biomimetic EV design strategies: (**B**) Nanoparticles wrapped by EV membrane (using EV membrane to wrap synthetic nanoparticles), (**C**) natural-artificial hybrid EVs (neural EVs recombined with synthetic or biological system components), (**D**) EV-mimicking nanoparticles (created using proteins and lipids to imitate the structure of natural EVs). Abbreviations: EV: extracellular vesicles. Figure created with BioRender.com

**Fig. 3:**
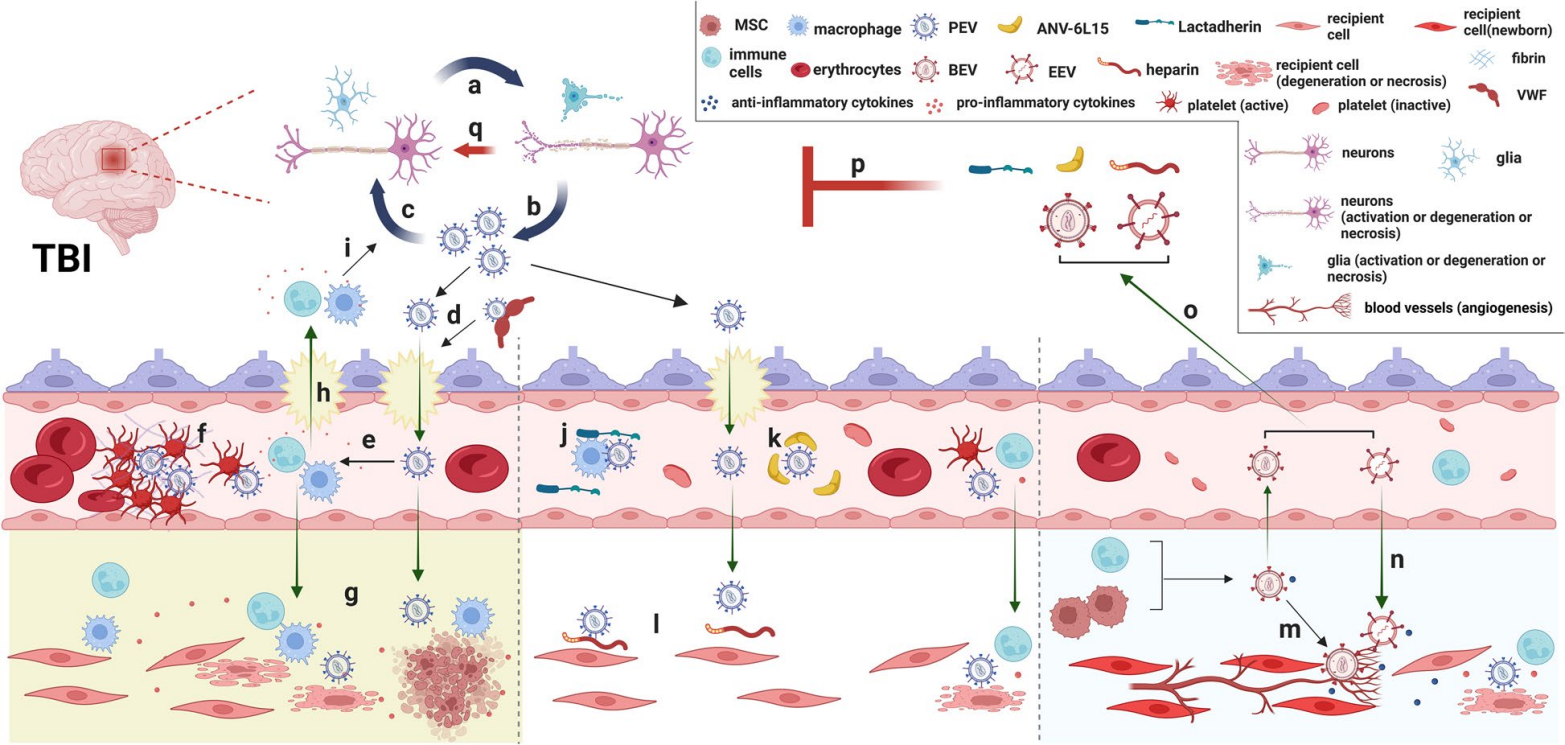
EV-based TBI treatment strategies: Neuronal cells appeared degenerative and necrosis (**a**) and released PEV (**b**) after TBI; PEV further exacerbate nerve cell damage (**a** & **c**); The combination of PEV and VWF into a complex significantly increased the permeability of the BBB and further damaged the normal structure of the BBB (**d**), so PEV entered the peripheral circulation through the damaged BBB; PEV in the peripheral circulation activated platelets and caused them to release more EVs, while EVs trigger and propagate the coagulation cascade, leading to of thrombosis and ultimately coagulopathy (**f**); Meanwhile, PEV in the circulation activate immune cells to trigger an inflammatory response (**e**). Activated immune cells not only could enter the brain parenchyma through the damaged BBB (**h**) and exacerbate nerve cell damage (**i**) and PEV release (**b**), but also synergized with PEV to cause degeneration and necrosis of recipient cells (**g**), eventually causing systemic complications; In addition, persistent inflammation and nerve damage increased the risk of neurodegenerative diseases (**a**, **b** & **c**). There are two strategies for EV-based TBI treatment. First, the function of PEV is inhibited, for example, accelerating the clearance of PEV by lactadherin (**j**), inhibiting the procoagulant function of PEV by ANV-6L15 (**k**) and inhibiting the uptake of PEV by recipient cells by heparin (**l**). Second, BEV, such as immune cell/MSC-EV (**m**) can be increased or EEV can be infused (**n**), which will play an anti-inflammatory role and promote tissue repair. Meanwhile, BEV and EEV can also carry protective factors through the BBB to the brain parenchyma to play a protective role (**o**). In conclusion, EV-based TBI treatment strategies can effectively prevent nerve cell damage and PEV release (**p**); Furthermore, BEV and EEV can also repair damaged nerve cells and promote nerve regeneration (**q**). Abbreviations: ANV-6L15: ANV-6L15 fusion protein; BBB: blood–brain barrier; BEV: biological extracellular vesicles; EEV: engineered special purpose extracellular vesicles; EVs: extracellular vesicles; MSC: mesenchymal stromal cells; PEV: pathological extracellular vesicles; TBI: Traumatic brain injury; VWF: von Willebrand factor. Figure created with BioRender.com

Direct modification of natural EVs (modified EVs) can significantly improve their delivery, ability to target, and therapeutic efficacies. Researchers have used DNA, RNA, and proteins as well as small-molecule drugs to modify EV membranes or cargo in order to achieve targeted therapeutics [17] (Fig. 2A and 3). To prevent the secondary damage induced by ischemic stroke, Tian et al. [158] conjugated c(RGDyK)-peptide to the membrane surface of EVs to target EVs specifically to ischemic brain tissue. They found that the membrane-modified EVs carrying curcumin strongly inhibited the inflammatory response and apoptosis in the ischemic area in a mouse model. Liang et al. [159] introduced miR-26a, which inhibits the migration and proliferation of liver cancer cells, into EVs by electroporation. Sonication and extrusion may serve as more efficient methods of delivering drugs into EVs than electroporation, as shown by Haney et al. [160]. They introduced catalase into EVs using different methods such as room temperature incubation, saponin permeabilization, cyclic freeze–thaw, sonication or extrusion, and found that catalase-carrying EVs efficiently accumulated in neurons and microglia in the brains of PD mice and exerted a potent form of neuroprotection [160]. However, these mechanical manipulations that allow for passive introduction of drugs into EVs may destroy the integrity of EV membranes and thus reduce their therapeutic effects. Therefore, inducing donor cells to actively uptake and carry drugs is a highly viable option for protecting the integrity of a drug-carrying EV membrane. In their research, Haney et al. generated drug-loaded MSC-derived EVs by co-incubating MSCs with paclitaxel [161]. This method is simple and feasible, and preserves the original information of EV structure, but it is not perfect either. It is only suitable for specific small-molecule drugs, and the efficiency of their introduction into EVs is low, so it cannot be used for large-scale production of drug-loaded EVs. In conclusion, further research is needed to elucidate the drug-loading capabilities of different EVs, enrich the catalog of loaded drugs, and standardize EV drug-loading protocols.

#### Biomimetic EVs

To solve the problems of low yield, complex and diverse preparation procedures, and poorly defined synthesis mechanisms of natural EVs, researchers have synthesized biomimetic EVs such as synthetic nanoparticles wrapped by EV membrane (Fig. 2B), natural-artificial hybrid EVs (combining natural EVs with other synthetic or biological components; Fig. 2C), EV-mimicking nanoparticles (using proteins and lipids to imitate the structure of natural EVs, Fig. 2D) [155]. In addition to the advantages of controllable preparation conditions, simple production procedures, high yield, and homogeneity, these biomimetic EVs also retain similar physical and chemical properties to natural EVs [17]. Furthermore, synthesized biomimetic EVs have also shown high drug loading [162, 163], more precise cell targeting properties [164–166] and fewer safety hazards [162] in preclinical studies. In conclusion, the successful production and application of biomimetic EVs have improved the drug-loading efficiency, targeting accuracy, and applicability of EEV, reduced the safety hazards of natural EVs, and paved the way for drug-loaded treatments that use EEV (Fig. 3).

## EVs are an emerging class of diagnostic markers for TBI and associated complications

The diagnosis and evaluation of TBI depend primarily on conventional neuroimaging techniques, such as Computer Tomography (CT) and Magnetic Resonance Imaging (MRI). These imaging techniques cannot identify microstructural damages [167] and provide less real-time information on the brain and other changes such as coagulation dysfunction, neuroinflammation, blood–brain barrier disruption, and excitotoxicity [168, 169]. A variety of emerging biomarker candidates to define TBI at cellular levels have been recently investigated [23, 24, 170], among them are EVs of different cells of origin (Table 3).

**Table 3:** EVs are an emerging class diagnostic markers for TBI and associated complications^a^

EV Sources: Cell type/Tissue/species	Method of measurement	Tested components of EV	Sampling time point	Main findings	Ref
Endothelial, platelet, and leukocyte/plasma/human	**Flow cytometry**	**•TF** **•P-selection**	**6,12,24,48 and 72 h post-injury**	**MP counts in cerebral vein samples, regardless of cell origin, were higher in TBI cases compared with healthy groups; and MP counts decreased sharply from high levels shortly after TBI to slightly higher levels 72 h later**	**M.Nekludov et al.** [54]
EV in circulating blood /Plasma/human	**Paramagnetic bead-based enzyme-linked immunosorbent assay**	**Protein quantification**	**years post injury**	**The levels of plasma EV and NfL were significantly increased in patients with repeated mild TBI; Even years after injury, the increase was greatest in patients with chronic post-concussive syndrome, post-traumatic stress disorder, and depression symptoms**	**Guedes VA et al.** [171]
BDEVs/plasm/mouse and human	**•RNA sequencing** **•Machine learning algorithms**	**miRNA**	**1 h after single or multiple injuries, 0.4–120 h after injury(clinical samples)**	**Machine learning algorithms processing miRNAs in brain-derived EVs can detect various injury types and characteristics of TBI, reflecting the heterogeneity of human TBI injury and recovery more accurately than traditional diagnosis**	**Ko J et al.** [172]
EV in circulating blood /plasm/human	**•ELISA** **•RNA Sequencing**	**•GFAP** **•Short noncoding RNA**	**—**	**Increased GFAP concentrations in EVs from TBI patients with altered consciousness, as well as differential expression of multiple miRNAs targeting TBI-related pathways, suggest that EVs may be potential carriers of TBI biomarkers**	**Puffer RC et al.** [173]
Astrocytes/culture medium/ human	**Droplet digital PCR**	**specific** **subset of miRNAs**	**24 h post-IL-1β induced inflammatory stress**	**Astrocyte-derived EVs express a specific subset of miRNA that may play a potential role in modulating inflammatory responses**	**Manoshi Gayen et al.** [34]
Neurons/ brain/ Mouse	**RNA sequencing**	**miR-21**	**1–7 days post- injury**	**As a potential neuron-derived EV cargo, miR-21 may mediate the activation of microglia**	**Harrison EB et al.** [35]
Brain endothelial cells/ plasma/Mouse	**•Flow Cytometry** **•Electron microscopy**	**Tight junction proteins**	**24 h post-injury**	**•Brain endothelial cells release eEVs containing TJP and endothelial markers to mediate vascular remodeling after TBI** **•Detection of brain endothelial-derived EVs provides a novel approach to assess BBB structure and function in trauma and neuroinflammation**	**Andrews AM et al.** [38]
Neurons and glial cells/ brain, plasma/Mouse	**•Flow Cytometry** **•Electron microscopy**	**PS and TF on membrane**	**0.5,1,3 and 6 h post-injury**	**•The traumatized brain releases procoagulant BDMPs into the circulation to trigger a disseminated coagulation cascade** **•The abundance of PS and TF on the membrane surface is responsible for the procoagulant activity of BDMPs**	**Ye Tian et al.** [23]
Neurons and glial cells/ plasma/ Mouse	**•Flow Cytometry** **•Electron microscopy**	**CL on mitochondrial membrane**	**0.25, 0.5,1,3,7,10,14 days post-injury**	**•The mtMP is a major subset of BDEVs** **•Abundant CL on the membrane surface is responsible for mtMPs-triggered coagulation dysfunction after TBI**	**Zilong Zhao et al.** [24]
EV in circulating blood /plasma/human	**RNA sequencing**	**Specific mRNA and lncRNA**	**—**	**Analysis of SEV and LEV cargoes suggests that RNA may serve as novel, readily accessible biomarkers for AD, PD, ALS, and FTD in the future**	**Sproviero et al. D** [47]

^a^*Abbreviations AD* Alzheimer's disease, *ALS* amyotrophic lateral sclerosis, *BBB* blood–brain barrier, *BDEV* brain-derived extracellular vesicles, *BDMPs* brain-derived microparticles, *CL* cardiolipin, *eEVs* endothelial-derived extracellular vesicles, *ELISA* enzyme-linked immunosorbent assay, *EVs* extracellular vesicles, *FTD* frontotemporal dementia *GFAP* glial fibrillary acidic protein, *LEV* large extracellular vesicles, *MP* microparticles, *NfL* neurofilament light, *PCR* polymerase chain reaction, *PD* Parkinson's disease, *PS* phosphatidylserine, *SEV* small extracellular vesicles, *TBI* traumatic brain injury, *TF* tissue factor, *TJP* tight junction proteins

EVs can be evaluated quickly and cost effectively in body fluids such as peripheral blood samples because they can be released through BBB [174] to the circulation and remain relatively stable over a long period of time in storage [175]. The analysis of EV cargo can provide pathophysiological information on cells, tissues, and organs (Table 3), regarding issues such as coagulopathy, neuroinflammation, immune responses, and tissue repairs, which together provide a more comprehensive picture of short and long term outcomes of patients with TBI [2, 13], including mild TBI that cannot be defined as accurately using conventional neuroimaging techniques [171]. EVs generated either from injured cells or produced through synthetic means could serve as delivery vehicles for the treatment of TBI-related neurological diseases [101]. Interestingly, as more and more proteins or nucleic acids are identified as potential biomarkers for the diagnosis, treatments, and prognosis of TBI, EVs can also provide a valuable platform for detecting and evaluating existing and new biomarkers [176]. Ko et al. [172] developed a microchip diagnostic technique to more comprehensively characterize TBI by detecting miRNAs in brain-derived EVs to delineate the heterogeneity of TBI injury and recovery more accurately in patients. Puffer et al. [173] demonstrated that GFAP, a glial cell-specific biomarker, significantly increases in plasma EVs of patients with altered consciousness after TBI. A key issue is the lack of standardized protocols for EV extraction, characterization, and classification in the literature, making comparison among different studies challenging [170, 177] due to the high heterogeneity of EVs [172]. Furthermore, the development of machine learning algorithms will prove critical to more efficient use of EVs in understanding the pathogenesis, severity, treatments, and outcome predictions of patients with TBI [13].

## EV-based treatment of TBI

EV-based therapy is increasingly recognized as a new approach in addition to the surgical and non-surgical treatments of TBI for their intrinsic biological activities and for being used as drug delivery vehicles (Fig. 3). As reported by Khan et al., EVs, especially exosomes, which are very small EVs secreted from activated cells, will not only contribute to the diagnosis of TBI, but will also play an important role in the personalized treatment of TBI patients [12].

### Eliminates the detrimental effects of PEV on TBI

Since PEV are released by parental cells at the time of TBI, potentially resulting in local and systemic pathologies [23, 24], removing or blocking pathological activities of these PEV is a primary therapeutic goal (Fig. 3). For example, EV-induced systemic coagulopathy can be prevented by preventing the assembly of tenase complex on the surface of EVs that express anionic phospholipids [25], removing EVs from the circulation [26], or blocking their adhesion to endothelial cells [37, 86]. Our study shows that the fusion protein ANV-6L15, which is a recombinant fusion protein that fuses the Kunitz protease inhibitor module 6L15 into a variant ANV of annexin V [178], blocks tenase assembly on EVs to prevent TBI- induced coagulopathy and improve outcomes of TBI in mouse models [25]. Furthermore, lactadherin (milk fat globule–epidermal growth factor 8 [MFGE-8]), which is a 41 to 46 kDa glycoprotein containing an N-terminal epidermal growth factor-like domain and two C-terminal discoidin domains (C1&C2) [179], can bind PS on EVs to remove them from the circulation by facilitating EV phagocytosis [26]. In addition, our previous work has also demonstrated that blocking the adhesion of PEV to endothelial cells can be achieved by enhancing VWF proteolysis or blocking its active site [37, 86]. Interestingly, Kerr et al. [91] reported that the anticoagulant enoxaparin (Lovenox) inhibits the uptake of PEV by target cells and thereby reduces EV-mediated activation of inflammasome in the brain and lungs of mice subjected to severe TBI, potentially by suppressing the internalization of EVs by target cells [180] (Fig. 3). Enoxaparin has also been shown to reduce the cerebral edema and promote neurological recovery of TBI mice [181], but it carries a high risk for secondary bleeding, especially in the brain [182, 183].

### Infusing BEV has beneficial effects on TBI

Use of BEV as therapeutic agents remains small in scale, including the use of MSC-derived EVs in a TBI setting [184]. MSCs are multipotent stem cells with self-renewal ability and differentiation potential [185]. They have emerged as TBI therapeutics [186, 187] to regulate neuroinflammation [188] and repair damaged nerves [189]. However, recent studies show that MSC-associated regeneration and repair are mediated by bioactive factors released by them [184, 190]. These bioactive factors can be packed in MSC-derived EVs [113, 128, 136–138]. The neuroinflammation-regulating activity of MSC-derived EVs is likely mediated through immune regulation to reduce the activation of microglia and macrophages and to increase anti-inflammatory cytokines while reducing pro-inflammatory cytokines in traumatically injured cerebral tissues [139, 140]. Micro RNAs packed in MSC-derived EVs are widely considered the key factors for these regulatory processes [141], including those inhibiting macrophages through Toll-like receptor signaling [191] and hypoxic inflammation by inhibiting hyperproliferative pathways such as hypoxia-induced STAT3-mediated signaling [142]. The miRNAs in MSC-derived EVs may also promote neurogenesis and angiogenesis. As key regulators of synaptic plasticity [192], miRNAs target transcription factors to regulate neurogenesis [193]. In vitro studies have shown that MSC-derived EVs deliver miR-124 and miR-145 to human neural progenitor cells and astrocytes, altering gene expressions in recipient neurons to increase neuronal differentiation [194], even though the delivery pathway remains to be mechanically defined.

As a classic subset of BEV, MSCs are the main player used by researchers to generate target EVs, which have achieved promising results in animal models (Fig. 3). The study of other potential cells still needs to be investigated to determine the most suitable source of BEV. Since the cargo of MSC-derived EVs is highly dependent on the type of MSCs as well as the surrounding microenvironment [143, 195], standardization of MSC sources and production conditions is necessary. In addition, it is important to achieve standardization of the isolation and characterization of MSC-derived EVs, as this involves screening for specific EVs. More importantly, the molecular mechanism by which MSC-derived EVs improve tissue repair remains poorly understood, and filling this knowledge gap may provide more definitive guidance to the therapeutic use of MSC-derived EVs in TBI.

### Design and clinical application of EEV in TBI in the future

The design and clinical application of EEV must take into account the potential effects of its structure and contents on recipients. As we have previously reported [23], infusion of PS^+^/TF^+^EV into uninjured mice has been shown to result in severe coagulopathy and severe vasospasm [28]. EVs carrying large amounts of PS and/or TF on their surface result in higher mortality in mice, regardless of whether the EVs contain any therapeutically valuable factors.

In addition, possible problems in the clinical translation of EEV in TBI should be considered. For example, how drug-loaded EEV be infused in the acute phase of TBI? One of the challenges here involves how to develop an appropriate and realistic EEV treatment plan in the short post-injury period. Further, what is the relationship between EEV treatment and neurosurgical treatment? The answers to these questions will determine the indications for EEV therapy.

## Conclusion

In summary, EV-based TBI treatment strategies should be based on several principles: eliminating or inhibiting the pathological effect of PEV to minimize their activities in causing secondary damage to TBI patients, while promoting the repair function of BEV or infusion of drug-loaded EEV to improve the prognosis of patients with TBI in a targeted manner. Clarifying the difference between PEV and BEV will pave the way for the construction of EEV and the diagnosis and treatment of TBI. Therefore, accelerating the proteome analysis of PEV and BEV is an urgent task. Enriching the database of PEV and BEV is helpful to identify the specific types and pathological processes of TBI, and the identification of the pathogenesis as well as structure and function of PEV and BEV will prove helpful for the clinical translation of EVs. This work will depend on more in vitro and in vivo experiments and multi-center clinical studies.

## Data Availability

Not applicable.

## Abbreviations

Aβ: Amyloid β-peptide
AD: Alzheimer's disease
α-syn: α-Synuclein
BBB: Blood–brain barrier
BDEVs: Brain-derived extracellular vesicles
BEV: Biological extracellular vesicles
CL: Cardiolipin
CNS: Central nervous system
CT: Computer Tomography
CTGF: Connective tissue growth factor
CX43: Connexin 43
DAMPs: Damage-associated molecular patterns
EEV: Engineered special purpose extracellular vesicles
eEVs: Endothelial cell-derived EVs
EVs: Extracellular vesicles
exMTs: Extracellular mitochondria
GJA1-20 k: Gap junction alpha 1 -20 kDa
HMGB1: High-mobility group box protein 1
ILVs: Intraluminal vesicles
LBs: Lewy bodies
LNs: Lewy neurites
MFGE-8: Milk fat globule–epidermal growth factor 8
MRI: Magnetic Resonance Imaging
MSCs: Mesenchymal stromal cells
MVEs: multivesicular endosomes
PAMP: pathogen-associated molecular patterns
PD: Parkinson's disease
PEV: Pathological extracellular vesicles
pEVs: platelet-derived EVs
PS: phosphatidylserine
ROS: reactive oxygen species
TBI: Traumatic brain injury
TBI-IC: traumatic brain injury induced coagulopathy
TDP-43: TAR DNA-binding protein of 43 kDa
TF: tissue factor
TGFβ1: transforming growth factor β1
TLR7/8: toll-like receptors 7/8
VWF: von Willebrand factor
